# Inferring Therapeutic Targets in *Candida albicans* and Possible Inhibition through Natural Products: A Binding and Physiological Based Pharmacokinetics Snapshot

**DOI:** 10.3390/life12111743

**Published:** 2022-10-30

**Authors:** Zarrin Basharat, Kanwal Khan, Khurshid Jalal, Sulaiman Mohammed Alnasser, Sania Majeed, Marium Zehra

**Affiliations:** 1 Jamil–ur–Rahman Center for Genome Research, Dr. Panjwani Center for Molecular Medicine and Drug Research, International Center for Chemical and Biological Sciences, University of Karachi, Karachi 75270, Pakistan; 2 Dr. Panjwani Center for Molecular Medicine and Drug Research, International Center for Chemical and Biological Sciences, University of Karachi, Karachi 75270, Pakistan; 3 HEJ Research Institute of Chemistry, International Center for Chemical and Biological Sciences, University of Karachi, Karachi 75270, Pakistan; 4 Department of Pharmacology and Toxicology, Unaizah College of Pharmacy, Qassim University, Buraydah 52571, Saudi Arabia

**Keywords:** *Candida albicans*, fructose-bisphosphate aldolase, CADD, dynamics simulation, pharmacokinetics, ADMET

## Abstract

Despite being responsible for invasive infections, fungal pathogens have been underrepresented in computer aided therapeutic target mining and drug design. Excess of *Candida albicans* causes candidiasis, causative of thrush and vaginal infection due to off-balance. In this study, we attempted to mine drug targets (*n* = 46) using a subtractive proteomic approach in this pathogenic yeast and screen natural products with inhibition potential against fructose-bisphosphate aldolase (FBA) of the *C. albicans.* The top compound selected on the basis of best docking score from traditional Indian medicine/Ayurvedic library was (4-Hydroxybenzyl)thiocarbamic acid, from the ZINC FBA inhibitor library was ZINC13507461 (IUPAC name: [(2R)-2-hydroxy-3-phosphonooxypropyl] (9E,12E)-octadeca-9,12-dienoate), and from traditional Tibetan medicine/Sowa rigpa was Chelerythrine (IUPAC name: 1,2-Dimethoxy-12-methyl-9H-[1,3]benzodioxolo[5,6-c]phenanthridin-12-ium), compared to the control (2E)-1-(4-nitrophenyl)-2-[(4-nitrophenyl)methylidene]hydrazine. No Ames toxicity was predicted for prioritized compounds while control depicted this toxicity. (4-Hydroxybenzyl)thiocarbamic acid showed hepatotoxicity, while Chelerythrine depicted hERG inhibition, which can lead to QT syndrome, so we recommend ZINC13507461 for further testing in lab. Pharmacological based pharmacokinetic modeling revealed that it has low bioavailability and hence, absorption in healthy state. In cirrhosis and renal impairment, absorption and plasma accumulation increased so we recommend further investigation into this occurrence and recommend high dosage in further tests to increase bioavailability.

## 1. Introduction

Mycobiota, like other microbiota, is an essential part of the human body and resides in the genitourinary tract, gastrointestinal tract, respiratory tract, skin, and the mucosal membrane covering the oral cavity [1]. Commensal mycobiota can act as pathobiont in compromised host immunity and under certain clinical conditions [2]. Fungal infections spread drastically over the past few decades, and annual fatalities from fungal infections are higher than individually from TB, HIV, malaria, or breast cancer [3]. In healthcare institutions, candidiasis is still the most frequent hospital-acquired fungal infection [4], and almost 0.25 million people suffer from invasive candidiasis every year [5]. *C. albicans* is of significant clinical importance as it is responsible for causing superficial to invasive candidiasis. Surgery (especially abdominal surgery), burns, long-term hospitalization in an intensive care unit, and earlier use of broad-spectrum antibiotics and immunosuppressive drugs have all been risk factors for invasive candidiasis. *C. albicans* is widespread, with an increasing prevalence of 18–25% in the last few decades [6]. The hyphal form and biofilm formation in *C. albicans* is associated with various virulence characteristics, including as adhesion and the release of hydrolases, and plays an important part in the infection process [7,8].

The rapid development of antifungal resistance against azole, echinocandin, polyene, and nucleoside analogs in *C. albicans* support the need for more effective and less toxic treatment strategies [9]. Resistance in *Candida* spp. can be acquired or natural. Since the *Candida* spp. do not share resistance mechanisms, acquired resistance develops due to antifungal selection pressure in the individual patient or, less frequently, horizontal transfer of resistant strains across patients. In population-based studies, echinocandin resistance has been observed in *C. albicans* infections. Echinocandin resistance is linked to a mutation in two hot spot regions of *FKS1* in *C. albicans*, both in *FKS1* and *FKS2* [10]. The increased number of infections and emergence of antifungal resistance in *Candida* spp. emphasizes the need to work on novel therapeutic techniques in order to combat these infections. For this purpose, computer aided drug design (CADD) can expedite the drug design process via virtual screening approach (structure or ligand aided) [11]. This approach has become fundamental to pre-clinical screening of molecules. Drug targets are prioritized and then screened against libraries of compounds. Target prioritization reduces targets to a manageable number and aids choice of unique or conserved targets, depending on the requirement [12]. The selected targets can then be studied in detail against the drug-like molecules. Among the utilized libraries of compounds, the ZINC database is used in around 31.2% of studies [13], while a large proportion use natural products. In the present study, we analyzed the reference genome of *C. albicans* and identified several drug targets. Natural product libraries of potent inhibitors were screened against it and dynamics simulation was performed to validate binding. Absorption, distribution, metabolism, excretion and toxicity (ADMET) profiling was also carried out for the top inhibitors. Apart from parameter inference, pharmacokinetic parameters were also simulated in the body compartments to determine bioavailability, plasma concentration, and absorption of the drug in a population group (*n* = 900 individuals), with a diseased and healthy set of people.

## 2. Material & Methods

### 2.1. Data Retrieval

The NCBI database was used for obtaining the genome sequence of reference strain SC5314 of *C. albicans*. Human proteome was retrieved from the Universal Protein Resource (UniProt) database to investigate and remove paralogs. Following the deletion of all paralogs, Database of Essential Genes (DEG) [14] and Cluster of Essential Genes (CEG) [15,16] were used for extracting/identifying critical genes. To find the druggable properties and drug targets of essential genes, the DrugBank database served the purpose.

### 2.2. Essentiality Analysis

The resulting core genome non-homologous sequences were analyzed and characterized through further downstream processing. Essentiality analysis was performed to identify essential genes [17] as excellent drug targets. Essential genes are necessary for the survival of organisms even in harsh conditions [14]. The importance of selecting essential genes as drug targets is that they restrain and hinder a pathogen’s proliferation, functionality, and pathogenicity. Database of Essential Genes (DEG) was used to further analyze the protein role of non-homologous genes. Amino acid sequences of these genes were BLASTed against DEG [18] with an E value of 10^−5^ [19]. Genes depicting high homology with DEG were analyzed through CEG (Cluster of Essential Gene database) based on alignment and functionality [15]. This clustering data helps refine data and reduce the chances of false positive results during an examination. The resultant genes given by both CEG and DEG were analyzed, and shared genes in both databases were selected for further processing.

### 2.3. Drug Target Mining

For the non-homology analysis of drug targets against the human host, the essential coding sequences were screened against the human genome using BLASTp [20]. This analysis is performed to prevent drug binding and decrease the chances of cross-reactivity of a drug. Protein sequences of targeted gene sets were subtracted from the human proteome data with a threshold value of 10^−2^. The standard gap penalty of 11 and the gap extension penalty of one were selected for estimation [21]. It is also crucial to find drug targets that are non-homologous to human gut flora. The resultant targets were screened against gut flora by BLASTp [22], subcellular localization was determined from CELLO server (http://cello.life.nctu.edu.tw/(accessed on 1 September, 2022)), and DrugBank dataset was aligned to check the therapeutic matches of targets.

### 2.4. Virtual Screening

Selected protein fructose-bisphosphate aldolase (FBA) was obtained from the Alpha fold server [23]. Structure preparation and screening was performed against Ayurvedic library (*n* = 2103 compounds), Sowa rigpa (*n* = 39 compounds), and ZINC inhibitor library of FBA (*n* = 1922 compounds), according to previously described protocol [24]. (2E)-1-(4-nitrophenyl)-2-[(4-nitrophenyl)methylidene]hydrazine was used as a control, as it inhibits this enzyme completely at 0.05 mM concentration, pH = 7, temperature = 37 C, Ki value = 0.0017 [BRENDA details at https://www.brenda-enzymes.org/literature.php?e=4.1.2.13&r=748282] (accessed on 10 September, 2022) [25].

Dynamics simulation was carried out using GROMACS for 100 ns [26,27]. Parameters were: Solvation using Simple Point Charge (SPC) water model; Energy minimization algorithm: Steepest descent; NVT and NPT ensemble: 50,000 steps; Pressure = 1 atm pressure, Temperature = 300 K.

### 2.5. ADMET Profiling

To determine pharmacokinetics and solubility, ADMET analysis was performed using PkCSM server (http://biosig.unimelb.edu.au/pkcsm/(accessed on 12 September, 2022)). This server uses graph modeling for representation of chemical entities, by intaking SMILE format for a compound [28]. The output is classified under five categories and obtained through a user-friendly webserver display.

Simulation of physiological pharmacokinetic parameters, leading to drug absorption and concentration determination of compound in plasma with reference to time was performed using GastroPlus (version 9.8.2, Simulation Plus, Inc., Lancaster, PA, USA). This software determines pharmacokinetics of a drug or formulation through body compartments [29,30]. We used oral administration of our prioritized compounds in 100 mg tablet composition, with 250 mL intake of water and simulation of a compartmental absorption and transit (ACAT) model through stomach, duodenum, jejunum, ileum, and colon for 10 h. We used the following parameters: physiology state = fasted, animal = human, particle radius = 25 microns, particle density = 1.2 g/mL, pH = 7.2, solubility value determined by method of Delane, precipitation = first order, paracellular model for jejunal permeability = Zhimin, nucleation model = diffusion, dissolution model for bile salt effect = Johnson, effective permeability calculated from permeability converter using the formula Peff = (10^−1.5383 + 0.811^ * ^log human_permeability^), clearance from the central compartment (assumed as general body clearance) CL = 0.142 L/h, central compartment volume Vc = 0.1 L/kg, first pass extraction for liver fixed at 68%, tissue vs. plasma time database for simulation in a population of 300 healthy, 300 cirrhotic, and 300 renally impaired individuals to elucidate unevenness in drug exposure. Parameters obtained were percentage of bioavailable drug, along with absorption in intestine and portal vein. Concentration–time curve integral was calculated (after a single dose), as this value can also help guide dosing for compounds with narrow therapeutic index.

## 3. Results

### 3.1. Therapeutic Candidate Mining

**Figure 1:**
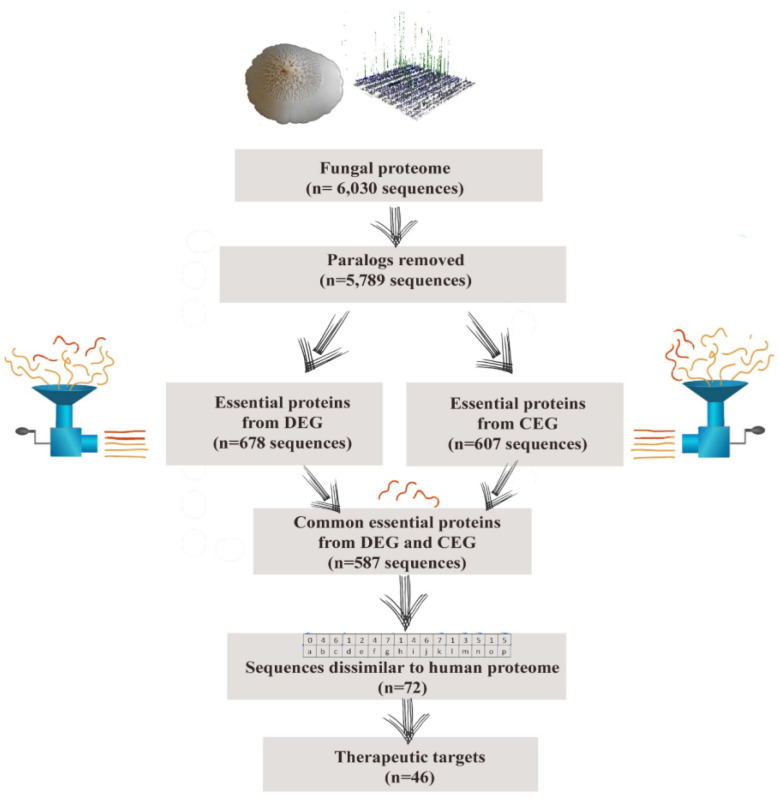
Hierarchal differential proteome analysis of *C. albicans* SC5314, showing number of sequences retained at each step of analysis.

*C. albicans* SC5314 has eight chromosomes, with a genome size of 14.3 Mb. Its total proteome comprises more than 6000 proteins (Figure 1). Proteome subtraction is a well-defined technique for therapeutic target mining. We utilized this method for inferring druggable proteins and obtained 46 hits (Table 1). Among these, FBA, commonly known as aldolase (EC number: 4.1.2.13), was selected for further processing. FBA has a key role in the glycolysis and gluconeogenesis of the *C. albicans*. Rodaki et al. have determined that it is essential for the growth of this yeast and is an attractive drug target as it is present for an essential pathway in this yeast but varies considerably from that of human aldolase [31]. FBA is present in copious amounts and has a quite stable structure. Three-dimensional coordinates of its protein structure were obtained from Alpha fold database, depicting two domains. It has an α/β domain, pleated into a TIM barrel, which consists of the active site. After necessary preparation in MOE, FBA was subjected to energy minimization. The prepared structure was subjected to structure-based docking for virtual screening of ligands.

### 3.2. Virtual Screening

**Figure 2:**
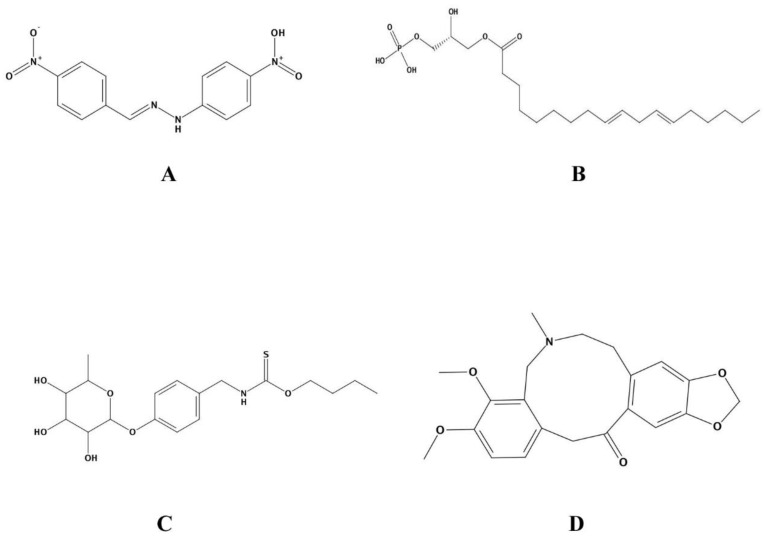
Two-dimensional structural depiction of (**A**) Control, (**B**) ZINC13507461, (**C**) (4-Hydroxybenzyl)thiocarbamic acid, and (**D**) Chelerythrine.

**Figure 3:**
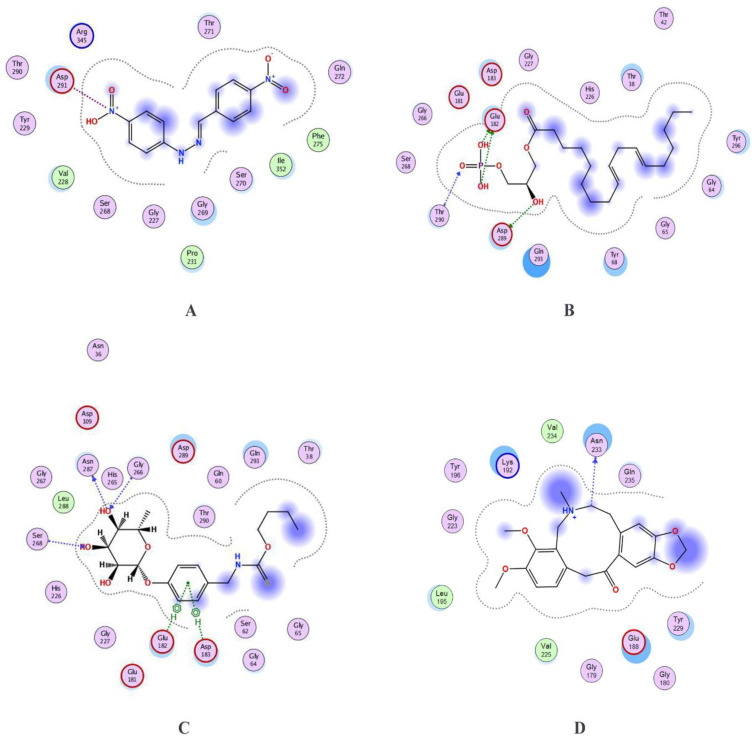
Two-dimensional interaction depiction between (**A**) FBA-Control, (**B**) FBA-ZINC13507461, (**C**) FBA-(4-Hydroxybenzyl)thiocarbamic acid, and (**D**) FBA-Chelerythrine. The purple region over ligand shows exposed residues. Violet circles indicate polar residues. Bluish circles over residues indicate exposed receptor areas.

Two natural product libraries were used for screening against FBA, comprising Ayurvedic and Sowa rigpa compounds. Apart from these, the inhibitor with best potency was obtained from BRENDA database and used as a control alongside the ZINC database compound classified as FBA inhibitors. Two-dimensional structures of the prioritized compounds are given in Figure 2. Among these, FBA made 14 interactions with control ((2E)-1-(4-nitrophenyl)-2-[(4-nitrophenyl)methylidene]hydrazine), including one acidic and one basic interacting residue (Figure 3). ZINC13507461 made 16, (4-Hydroxybenzyl)thiocarbamic acid made 21, and Chelerythrine made 11 interactions with four, five, and one acidic residue of FBA, respectively. Thr290 and Ser268 were conserved in making interactions in the control, ZINC13507461 and (4-Hydroxybenzyl)thiocarbamic acid, while Tyr229 was conserved in Chelerythrine and control. Apart from hydrogen bonding, other interactions were seen among complexes (Table 2). Ionic and covalent bonds are stronger than hydrogen bonds and the FBA-control complex depicted an ionic interaction, compared to hydrogen and pi-bonding between FBA-(4-Hydroxybenzyl)thiocarbamic acid and FBA-Chelerythrine, respectively. MM/PBSA values were lowest for FBA-control complex but for the individual ligand, it was least for the ZINC13507461.

**Figure 4:**
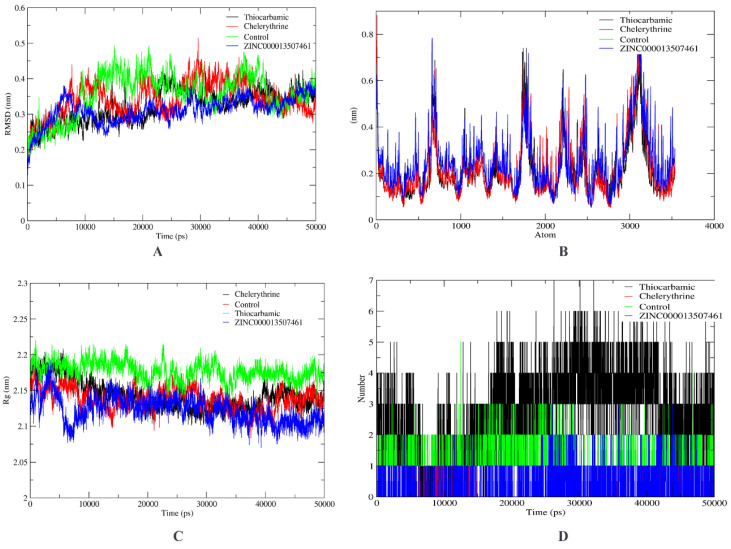
MD simulation depicting (**A**) RMSD, (**B**) RMSF, (**C**) radius of gyration, and (**D**) number of hydrogen bonds of the complexes.

MD simulation analysis revealed that the RMSD of the studied compounds did not exceed 0.5 nm/5 Å on the average (Figure 4). This shows that binding is fine. RMSF was 0.3 nm/6 Å on the average but there was very large deviation around atomic positions 700, 1800, 2200, 2400, 2600, and 3000–3200. Compared to the control, the radius of gyration of natural products was lower, showing a more compact/tight packing of the complexes. The highest number of hydrogen bonds was observed for thiocrabamic complex, depicting electrostatic interaction among complex atoms, followed by the control. However, the largest retention of hydrogen bonds of the ZINC13507461 complex was observed throughout the simulation time, while this was lowest for (4-Hydroxybenzyl)thiocarbamic acid.

### 3.3. ADMET Profiling

None of the prioritized compounds or control were substrates of CYP2D6, CYP2C9, or CYP3A4, predicted to cross blood-brain barrier, or hERG I inhibitors. Binding cytochrome enzymes leads to detoxification and excretion of the drug from the body. All of them bound to at least one cytochrome enzyme, except (4-Hydroxybenzyl)thiocarbamic acid. Only Chelerythrine was predicted to be a renal OCT2 substrate and hERG II inhibitor, with highest total clearance (Table 3). OCT binding can lead to renal clearance but inhibition of hERG leads to QT syndrome development so such a compound is not recommended. Control, but none of the screened compounds, showed AMES toxicity. Skin was non-sensitive to control and all three prioritized compounds. Hepatotoxicity was only shown by (4-Hydroxybenzyl)thiocarbamic acid. In light of these parameters, ZINC13507461 is recommended for further testing.

ADME was also simulated in the human body using a multiple compartment model in a group of 900 individuals. All compounds showed high intestinal and portal vein absorption, except ZINC13507461 (Table 4). Although it fulfilled many parameters, its bioavailability was relatively small at a concentration of 100 mg and it affected all parameters, such as plasma concentration and area under the curve (AUC). It is recommended that a higher dose be administered in test models to find its maximum potency range. However, its bioavailability and subsequently, plasma concentration, was higher in diseased state compared to non-cirrhotic and non-impairment of renal system. This tallies with the idea that elimination is not occurring effectively and drug is accumulating in plasma.

## 4. Discussion

Fungi are present in all environmental niches and several of their species are responsible for impacting human health [32]. Globally, fungal infections have a significant effect on human health. Over a quarter of the global population may have a fungal infection of the skin; 75% of women may have vulvovaginal candidiasis, and over a million individuals lose their lives annually due to invasive fungal infections [32,33]. Those with systemic fungal infections have an unacceptably high mortality rate, sometimes exceeding 50%. This is due to the fact that fungal infections are notoriously hard to identify and cure [34]. More precise diagnostics, safer and more effective antifungal medicines, and host-directed therapy are desperately needed in healthcare.

It has been observed that in immunocompromised and hospitalized patients, the death rate from bloodstream infections caused by *Candida* species is as high as 40–60% [35,36], where *C. albicans* continues to be the leading cause of life-threatening systemic candidiasis. It has the ability to switch back and forth between its yeast, pseudo hyphal, and hyphal development phases, making it a polymorphic organism [37]. Due to widespread usage of antifungals, *C. albicans* is developing drug resistance, which threatens antifungal treatment. This is why effective antifungal medicines with new pharmacological targets are required [38]. Humans share metabolic pathways and key cellular machinery, making fungal-selective targeting inadequate [7], but the whole-genome sequencing of the pathogens and advent of bioinformatics has opened up new paths, such as comparative subtractive genomics, to screen novel treatment and drug candidates [39]. In the current study a subtractive proteomic approach was applied to identify potential therapeutic targets in *C. albicans*. The approach has previously been successfully employed in prioritizing and designing drug targets against *Candida auris* [40]. Out of >6000 proteins, 46 potential drug targets were prioritized, and fructose-bisphosphate aldolase (FBA) was selected for further analysis. FBA is responsible for maintaining the glycolysis process by catalyzing fructose-1,6-bisphosphate (FBP) into dihydroxyacetone phosphate (DHAP) and D-glyceraldehyde-3-phosphate (G3P) [41]. Since FBA is not present in humans and crucial for its survival, it is an attractive target for the discovery of novel therapeutic candidates that selectively inhibit FBA. Amorim et al. also emphasized on the selectivity of FBA as a potential target against *C. albicans* [41]. It has been explored as a potential candidate for vaccine designing against *Candida glabrata* [42] and drug target against various fungal infections [43,44].

Antifungal medicines are confined to three primary classes: polyenes, which bind fungal cell membrane ergosterol; azoles, which impede ergosterol production; and echinocandins, which inhibit fungal (1,3)-β-D-glucan cell wall development. Echinocandins are harmless, however itraconazole, voriconazole, and amphotericin B are toxic [45]. The use of natural products as a source of active compounds in drug development has also received considerable attention. Roughly a hundred experimental natural products, many of them intended to combat cancer or bacteria, are now under human trials. Even before the advent of high throughput screening in the postgenomic era, natural products accounted for over 80% of all medications [46].

Insights into therapeutic repertoires for specific disease classes, medicine compounding principles, and chemical and pharmacological transformations used can be gained by comparing and contrasting the materia medica of various traditions, such as Indian and Thai Ayurveda, traditional Chinese medicine, Unani, and other Greco-Arabic traditions [47]. We utilized compound libraries of traditional Indian and Tibetan origin in this study, alongside the synthetic compounds reported as inhibitors of FBA. With time, compounds from traditional medicine are gaining ground and headed to the mainstream market. India has ample plant and herbs with medicinal properties (>3000 types) and coupled with traditional medicinal information, these are being actively pursued for complementary medicine or drug development resources [48]. Tibetan medicine is also an untapped resource and has been utilized since long ago, for the prevention and cure of numerous diseases [49]. Recently, randomized controlled clinical trials of medicines from these natural resources have been explored [50,51,52,53]. In the case of bacterial or fungal infections, plant-based extracts or oils have been used as antimicrobials and antifungals [54,55]. Combination of plant oil with antifungals for synergistic impact has also yielded very good results [56]. The bioactive compounds in these oils must have had good inhibition potential against the pathogens. This warrants further exploration using in silico and biophysics approaches.

In order to explore a drug’s action using CADD, one of the most crucial methods is structure-based drug discovery. Through the application of physics-based equations to determine the binding affinities of the compounds under test, various software examines the interaction between the compounds and the binding site [57]. These days, molecular docking and molecular dynamics are only two examples of the potential computational drug design methodologies being used to find novel drug ideas [58]. We utilized the compound structure information for these resources against the selected fungus and analyzed the binding computationally. New inhibitors were screened against the FBA target from natural product libraries using the biophysics approach. Consequently, three compounds (one from each library) were shortlisted, i.e., (4-Hydroxybenzyl)thiocarbamic acid (traditional Indian medicine/Ayurvedic library), ZINC13507461 (ZINC FBA inhibitor library), and Chelerythrine (traditional Tibetan medicine/Sowa rigpa) compared to the control (2E)-1-(4-nitrophenyl)-2-[(4-nitrophenyl)methylidene]hydrazine. As we previously predicted, (4-Hydroxybenzyl)thiocarbamic acid has anti-cancer potential targeting DNA repair pathway [59]. Chelerythrine is a potent and specific inhibitor of protein kinase C, with pharmacological actions including anticancer, antibiosis, and anti-inflammatory impact [60,61]. The results of MD simulation study showed that the average RMSD of the investigated compounds was 0.5 nm/5 Å. Thiocrabamic complex, which represents the electrostatic interaction between complex atoms, had the highest number of hydrogen bonds visible, followed by the control. While this was lowest for (4-Hydroxybenzyl)thiocarbamic acid, the ZINC13507461 complex showed the greatest retention of hydrogen bonds throughout the simulation.

Additionally, the ADMET profiling of these shortlisted compounds showed that all compounds possess no Ames test toxicity, none were substrates of CYP2D6, CYP2C9, or CYP3A4, predicted to cross blood-brain barrier, or hERG I inhibitors. Moreover, the systemic pharmacokinetics, ADME profiling and simulation in the human body using a central compartment model was performed. Since 4-(Hydroxybenzyl)thiocarbamic acid showed hepatotoxicity, while Chelerythrine depicted hERG inhibition, which can lead to QT syndrome, we recommend ZINC13507461 for further laboratory testing.

Physiologically based pharmacokinetic models (PBPK) describe the entire body physiology using connected equations and model parameters such as blood flow rates and tissue volumes. Since most drugs are administered orally, GI absorption PBPK models are crucial. These models can scale in vitro drug absorption, distribution, metabolism, and excretion data to in vivo scale. PBPK models are more accurate than allometry [62,63,64]. Jones et al. also validated the predicted plasma profiles in fed and fasted individuals for six different compounds included biorelevant solubility data into the GastroPlus^TM^ absorption model [65]. More subsequent investigations have proven the significance of this method [66], therefore, we recommend its usage in subsequent drug design and screening studies against pathogens. Our findings indicate that except for ZINC13507461, all of the compounds had very good absorption via the intestinal and portal veins. Therefore, high amounts of drug should be administered to test models to determine its optimal dosage. We recommend further tweaking of parameters, model training on more clinical data, and then altering conditions of age, enzyme kinetics, etc. to gain further insights into PBPK of the compounds.

## 5. Conclusions

*C. albicans* is the most common type of fungus found in the human microbiome, and it colonizes the body without causing any symptoms having impact on people’s health continues to be a worrying public health issue. The comparative investigations have demonstrated that *C. albicans* genomic structure enables response to a variety of environmental conditions and increases challenges for treatment. *C. albicans’* pathogenicity factors and processes span a broad spectrum, including dimorphism, biofilm development, thigmotropism, adhesion protein expression, and extracellular hydrolytic enzyme production. It is important that we find natural product mediated inhibitors against this pathogen. This work is a step towards this aim and drug target mapping as well as inhibition of FBA shows the potential of informatics assay for designing novel anti-fungal compounds against *C. albicans*. Previously, most studies have been limited to bacterial or viral pathogens due to their small genome size and ease of handling by computer. Here, a swift approach for examining natural products against the target through pharmacoinformatics exploration of medicinal compounds is undertaken, which can be replicated in other fungal pathogens. Safety of the compounds was endorsed by ADMET and physiological based pharmacokinetic simulation in the body shed light on dosing and relevant parameters. PBPK simulation is a comprehensive strategy for dosing and risk assessment as it renders anatomical account of the drug in body compartments, through mathematical modeling of complex ADME process. Our pipeline for CADD in *C. albicans,* is therefore a comprehensive computational strategy for finding bioactive natural drug-like compounds against the fungus. However, we suggest that experimental study is conducted on the compounds in mouse or other humanized models and cell lines, before proceeding for trials, to increase the effectiveness of anticipated target and our computational methodology.

## Figures and Tables

**Table 1:** Shortlisted drug targets from the proteome of *C. albicans* SC5314.

S. No.	Protein Accession Number	Protein Name	Protein Length	DrugBank Alignment Length	E-Value	Subcellular Localization
1	XP_019330652.1	lumazine synthase	206	157	1.33396 × 10^−29^	Cytoplasmic
2	XP_019330750.1	3-deoxy−7-phosphoheptulonate synthase	371	356	5.95566 × 10^−127^	Cytoplasmic
3	XP_019330821.1	trehalose 6-phosphate synthase/phosphatase complex subunit	1007	383	1.09471 × 10^−38^	Nuclear/Plasma membrane
4	XP_019331058.1	anthranilate synthase	522	410	3.47489 × 10^−58^	Cytoplasmic
5	XP_019331115.1	Bgl22p	924	358	1.55548 × 10^−12^	Cytoplasmic
6	XP_710092.2	4-amino-4-deoxychorismate synthase	822	461	1.1003 × 10^−36^	Nuclear
7	XP_710211.2	bifunctional chorismate synthase/riboflavin reductase [NAD(P)H]	413	376	1.39864 × 10^−52^	Mitochondrial/Nuclear
8	XP_710312.1	tryptophan synthase	702	394	1.85163 × 10^−144^	Cytoplasmic
9	XP_710700.2	pantoate--beta-alanine ligase	316	305	1.03048 × 10^−68^	Nuclear
10	XP_710729.1	3-deoxy−7-phosphoheptulonate synthase	370	353	3.03778 × 10−117	Cytoplasmic/Nuclear
11	XP_711703.1	hypothetical protein CAALFM_CR05750WA	342	129	5.37008 × 10^−0.8^	Cytoplasmic
12	XP_711706.1	alpha, alpha-trehalose-phosphate synthase (UDP-forming) TPS1	478	472	3.18455 × 10^−92^	Cytoplasmic
13	XP_712232.1	isocitrate lyase 1	550	250	2.64563 × 10^−44^	Peroxisomal
14	XP_713033.1	sulfonate dioxygenase	386	289	1.79648 × 10^−24^	Nuclear/Cytoplasmic
15	XP_713320.2	trifunctional histidinol dehydrogenase/phosphoribosyl-AMP cyclohydrolase/phosphoribosyl-ATP diphosphatase	838	424	9.87896 × 10^−114^	Cytoplasmic
16	XP_713806.1	hypothetical protein CAALFM_C111290WA	369	261	4.84039 × 10^−35^	Cytoplasmic
17	XP_714207.2	trifunctional dihydropteroate synthetase/dihydrohydroxymethylpterin pyrophosphokinase/dihydroneopterin aldolase	829	788	3.71427 × 10^−161^	Nuclear/Cytoplasmic
18	XP_714543.2	hypothetical protein CAALFM_C209810CA	434	400	1.30711 × 10^−42^	Cytoplasmic
19	XP_714705.1	hypothetical protein CAALFM_C305640WA	425	308	2.28143 × 10^−35^	Cytoplasmic
20	XP_714872.2	Dqd1p	146	124	4.79446 × 10^−33^	Cytoplasmic
21	XP_715352.2	uroporphyrinogen-III C-methyltransferase	561	418	2.52115 × 10^−42^	Nuclear/Cytoplasmic
22	XP_715357.1	Ebp7p	392	385	3.40361 × 10^−70^	Cytoplasmic
23	XP_715408.1	anthranilate phosphoribosyltransferase	369	313	2.82806 × 10^−41^	Cytoplasmic
24	XP_715440.2	Oye32p	432	379	4.31391 × 10^−35^	Cytoplasmic
25	XP_715739.1	dihydroorotase	358	356	4.16856 × 10^−54^	Cytoplasmic
26	XP_716238.1	hypothetical protein CAALFM_CR08310CA	385	286	2.24051 × 10^−36^	Nuclear
27	XP_716751.1	Hypothetical protein CAALFM_C601400WA	676	419	5.7776 × 10^−14^	Plasma membrane
28	XP_717003.2	Nik1p	1081	227	7.42174 × 10^−20^	Nuclear/Cytoplasmic
29	XP_718052.2	Ymx6p	622	304	4.11558 × 10^−0.6^	Plasma membrane
30	XP_718069.2	phenylacrylic acid decarboxylase	229	185	7.40789 × 10^−68^	Plasma membrane
31	XP_718219.1	5-methyltetrahydropteroyltriglutamate-homocysteine S-methyltransferase	775	767	0	Cytoplasmic
32	XP_718255.2	dethiobiotin synthase	212	205	7.64158 × 10^−16^	Chloroplast/cytoplasmic
33	XP_718258.2	biotin synthase	374	323	1.05435 × 10^−100^	Mitochondrial
34	XP_719019.1	3-methyl-2-oxobutanoate hydroxymethyltransferase	309	262	1.03531 × 10^−55^	Mitochondrial
35	XP_719048.1	2-isopropylmalate synthase	579	603	5.36732 × 10^−170^	Cytoplasmic
36	XP_719116.2	L-methionine (R)-S-oxide reductase	175	134	2.56642 × 10^−30^	Cytoplasmic
37	XP_721010.2	trifunctional hydroxymethylpyrimidine kinase/phosphomethylpyrimidine kinase/thiaminase	548	273	2.42294 × 10^−26^	Cytoplasmic
38	XP_721446.1	pyridoxine biosynthesis protein	292	285	9.51495 × 10^−106^	Cytoplasmic
39	XP_721536.1	trehalose-phosphatase	888	385	1.08126 × 10^−54^	Cytoplasmic
40	XP_721716.2	hypothetical protein CAALFM_C302070CA	388	287	5.53839 × 10^−34^	Cytoplasmic
41	XP_721934.1	ATP phosphoribosyltransferase	298	298	3.46987 × 10^−33^	Cytoplasmic
42	XP_721932.2	riboflavin synthase	237	219	5.41917 × 10^−35^	Cytoplasmic
43	XP_722690.1	fructose-bisphosphate aldolase	359	343	2.15136 × 10^−129^	Cytoplasmic
44	XP_722769.2	Aro1p	1551	430	2.38324 × 10^−70^	Cytoplasmic
45	XP_723161.2	trifunctional fatty acid synthase sub-unit	1884	763	2.04704 × 10^−107^	Cytoplasmic
46	XP_723517.1	Mts1p	513	290	3.95336 × 10^−26^	Plasma membrane

**Table 2:** Bonding interactions at atomic scale for the studied complexes. MM/PBSA value of the FBA protein was −25.79.

	Molecular Formula	Ligand Atom and Its Position	Receptor Atom/Residue	Interaction Type	Distance (Å)	Energy (Kcal/mol)	MM/PBSA Value of Complex	MM/PBSA Value of Ligand
Control	C_13_H_10_N_4_O	N29	OD1/ASP291	ionic	2.92	−5.0	−25.68	0.24
ZINC13507461	C_21_H_39_O_7_P	O57	OD2/ASP289	H-donor	3.25	−1.7	−25.50	−0.26
		O65	OE2/GLU182	H-donor	2.99	−5.3		
		O67	OE1/GLU182	H-donor	2.87	−7.1		
		O64	N/THR290	H-acceptor	3.61	−0.7		
(4-Hydroxybenzyl)thiocarbamic acid	C_8_H_9_NO_2_S	O14	O ASN287	H-donor	2.88	−0.7	−25.57	−0.03
		O12	N/SER268	H-acceptor	2.97	−2.2		
		O12	OG/SER268	H-acceptor	3.11	−0.6		
		O14	N/GLY266	H-acceptor	2.97	−0.9		
		6-ring	CA/GLU182	pi-H	3.92	−0.7		
		6-ring	N/ASP183	pi-H	4.61	−0.9		
Chelerythrine	C_21_H_18_NO_4_^+^	C8	O/ASN233	H-donor	3.38	−0.6	−25.64	0.11

**Table 3:** ADMET parameters of the studied compounds using pkCSM server, which uses graph-based prediction for toxicity and pharmacokinetic parameter estimation.

Property	Model Name	Unit	Predicted Value for Control	Predicted Value for ZINC13507461	Predicted Value for (4-Hydroxybenzyl)thiocarbamic Acid	Predicted Value for Chelerythrine
Absorption	Water solubility	Numeric (log mol/L)	−3.655	−4.445	−2.833	−3.123
	Caco2 permeability	Numeric (log Papp in 10^−6^ cm/s)	0.222	0.521	0.41	1.429
	Intestinal absorption (human)	Numeric (% absorbed)	86.122	59.414	57.252	96.43
	Skin permeability	Numeric (log Kp)	−2.766	−2.702	−3.041	−2.946
	P-glycoprotein substrate	Categorical (Yes/No)	Yes	Yes	Yes	No
	P-glycoprotein I inhibitor	Categorical (Yes/No)	No	Yes	No	Yes
	P-glycoprotein II inhibitor	Categorical (Yes/No)	No	Yes	No	Yes
Distribution	VDss (human)	Numeric (log L/kg)	0.531	−0.866	−0.716	0.53
	Fraction unbound (human)	Numeric (Fu)	0.188	0.151	0.3	0.311
	BBB permeability	Numeric (log BB)	−0.513	−1.571	−1.302	0.025
	CNS permeability	Numeric (log PS)	−2.332	−3.099	−4.217	−2.16
Metabolism	CYP3A4 substrate	Categorical (Yes/No)	No	Yes	No	Yes
	CYP1A2 inhibitor	Categorical (Yes/No)	Yes	No	No	No
	CYP2C19 inhibitor	Categorical (Yes/No)	Yes	No	No	Yes
	CYP2D6 inhibitor	Categorical (Yes/No)	No	No	No	Yes
Excretion	Total clearance	Numeric (log ml/min/kg)	0.354	0.453	0.154	0.879
Toxicity	Max. tolerated dose (human)	Numeric (log mg/kg/day)	0.071	0.079	0.848	0.095
	Oral rat acute toxicity (LD50)	Numeric (mol/kg)	2.513	2.985	3.023	3.411
	Oral rat chronic toxicity (LOAEL)	Numeric (log mg/kg_bw/day)	2.178	2.733	2.966	1.692
	*T. Pyriformis* toxicity	Numeric (log ug/L)	0.598	0.292	0.271	0.333
	Minnow toxicity	Numeric (log mM)	1.733	−1.682	3.117	0.78

**Table 4:** Pharmacokinetic parameters of the studied compounds presented as their mean values. Cmax is the maximum plasma concentration in central compartment at end point of simulation, Tmax is the time when Cmax is attained, AUC(0-inf) is area under the central compartment plasma concentration–time curve which can be extrapolated to infinity, AUC(0-t) is area under the central compartment plasma concentration–time curve for the time of simulation (i.e., 10 h).

Condition	Compounds	Intestinal Absorption of Compound Fa (%)	Portal Vein Absorption of Compound FDp (%)	Bioavailable Drug F (%)	Cmax (µg/mL)	Tmax (h)	AUC(0-inf) (ng-h/mL)	AUC(0-t) (ng-h/mL)
Healthy	Control	81.626	80.078	25.145	3.3904	9.7593	1,034,000	27,820
	ZINC13507461	11.461	10.906	3.6584	0.4671	10	2622.2	2622.2
	Chelerythrine	99.582	99.42	31.551	4.3333	2.5876	226,700	38,260
	(4-Hydroxybenzyl)thiocarbamic acid	79.27	77.024	24.757	2.9855	8.8793	431,900	23,550
Cirrhosis	Control	82.783	80.784	80.784	5.6819	9.8013	4,499,000	47,250
	ZINC13507461	11.565	11.025	11.025	1.0741	10	5728	5728
	Chelerythrine	99.903	99.866	99.866	0.8644	0.865	26,090,000	5852.2
	(4-Hydroxybenzyl)thiocarbamic acid	78.662	76.185	76.185	2.2192	9.9329	16,710	16,710
Renal impairment	Control	82.792	80.896	80.896	4.9496	9.916	1,215,000	40,110
	ZINC13507461	11.694	11.148	11.148	1.0993	10	5819.1	5819.1
	Chelerythrine	99.645	99.487	31.45	4.2081	2.6187	225,600	37,180
	(4-Hydroxybenzyl)thiocarbamic acid	79.022	76.616	25.489	3.1081	8.8371	839,000	24,430

## Data Availability

Data is derived from a source in the public domain (Genbank accession no: CP000792.2) and is incorporated into the article.
